# Reduction of BSI associated mortality after a sepsis project implementation in the ER of a tertiary referral hospital

**DOI:** 10.1038/s41598-023-31219-1

**Published:** 2023-03-29

**Authors:** Elena Seminari, Marta Colaneri, Marta Corbella, Annalisa De Silvestri, Alba Muzzi, Stefano Perlini, Ilaria Francesca Martino, Lea Nadia Marvulli, Alessia Arcuri, Marcello Maffezzoni, Rita Minucci, Enrica Bono, Patrizia Cambieri, Piero Marone, Raffaele Bruno

**Affiliations:** 1 Clinica di Malattie Infettive, Fondazione IRCCS Policlinico San Matteo, Piazzale Golgi 2, 27100 Pavia, Italy; 2 UOC Microbiologie e Virologia, Fondazione IRCCS Policlinico San Matteo, Pavia, Italy; 3 Servizio di Epidemiologia Clinica e Biometria Direzione Scientifica, Fondazione IRCCS Policlinico San Matteo, Pavia, Italy; 4 Direzione Medica di Presidio, Fondazione IRCCS Policlinico San Matteo, Pavia, Italy; 5 Emergency Medicine Unit and Emergency Medicine Postgraduate Training Program, Fondazione IRCCS Policlinico San Matteo, Pavia, Italy; 6 Department of Clinical, Surgical, Diagnostic and Pediatric Sciences, Fondazione IRCCS Policlinico San Matteo, University of Pavia, Pavia, Italy

**Keywords:** Infectious diseases, Bacterial infection, Microbiology, Medical research

## Abstract

The emergency room (ER) is the first gateway for patients with sepsis to inpatient units, and identifying best practices and benchmarks to be applied in this setting might crucially result in better patient’s outcomes. In this study, we want to evaluate the results in terms of decreased the in-hospital mortality of patients with sepsis of a Sepsis Project developed in the ER. All patients admitted to the ER of our Hospital from the 1st January, 2016 to the 31stJuly 2019 with suspect of sepsis (MEWS score ≥ of 3) and positive blood culture upon ER admission were included in this retrospective observational study. The study comprises of two periods: Period A: From the 1st Jan 2016 to the 31st Dec 2017, before the implementation of the Sepsis project. Period B: From the 1st Jan 2018 to the 31stJul 2019, after the implementation of the Sepsis project. To analyze the difference in mortality between the two periods, a univariate and multivariate logistic regression was used. The risk of in-hospital mortality was expressed as an odds ratio (OR) and a 95% confidence interval (95% CI). Overall, 722 patients admitted in ER had positive BC on admissions, 408 in period A and 314 in period B. In-hospital mortality was 18.9% in period A and 12.7% in period B (p = 0.03). At multivariable analysis, mortality was still reduced in period B compared to period A (OR 0.64, 95% CI 0.41–0.98; p = 0.045). Having an infection due to GP bacteria or polymicrobial was associated with an increased risk of death, as it was having a neoplasm or diabetes. A marked reduction in in-hospital mortality of patients with documented BSI associated with signs or symptoms of sepsis after the implementation of a sepsis project based on the application of sepsis bundles in the ER.

## Introduction

Sepsis is still a major cause of death. Since the emergency room (ER) is the first gateway for patients with sepsis, identifying best practices to be applied might crucially result in better patients’ outcomes.

Although some shadows have been cast on the efficacy of sepsis bundles implementation^1,2^, others widely support its value^3,4^. The bundles have been demonstrated to improve survival, through modification of the physician’s clinical approach, and quality improvement^5,6^. As happens with poly-trauma, acute myocardial infarction and stroke^7^, also in the sepsis case, bundles application might avoid discontinuous and delayed treatments, by providing a standardised approach for timely and appropriate management of patients^8^.

Although some studies found an association between timing of antibiotics and mortality in septic patients^9,10^, results from other studies have failed to observe it ^11,12^.

**Figure 1:**
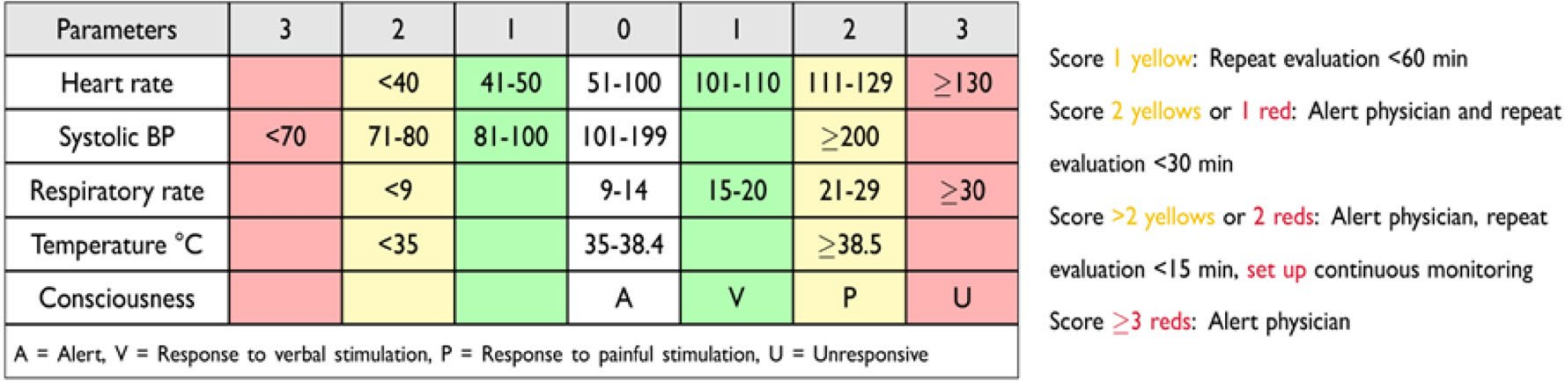
Table for assignment of patient priority code at the ER triage.

**Figure 2:**
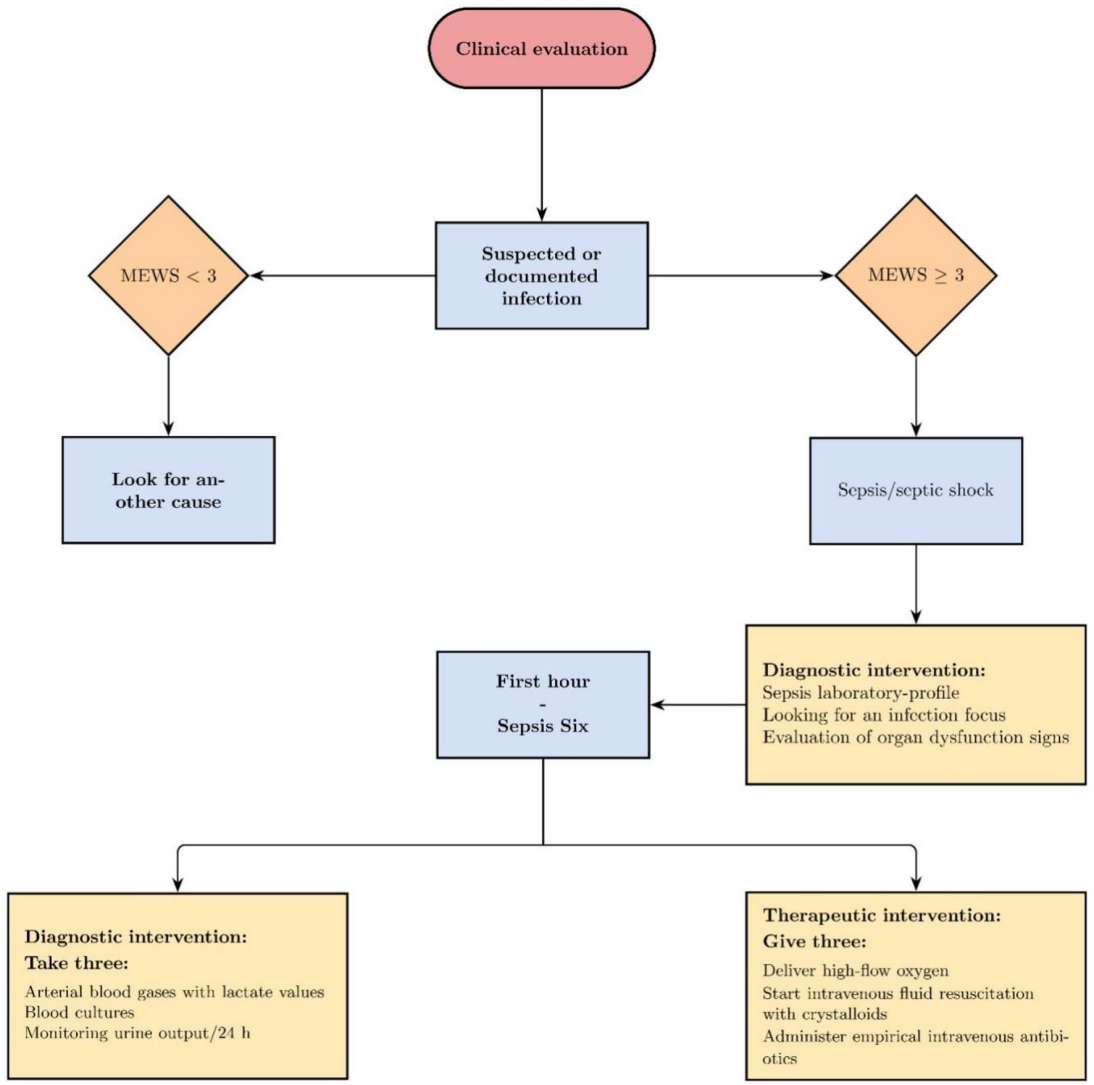
Patient management flow chat at the ER. *MEWS* modified early warning score. First hour—“sepsis six” box is referred to the 3 diagnostic (take three) and 3 therapeutic (give three) steps to be delivered by staff within 1 h.

Our objective was to retrospectively evaluate the results of a broader project developed in the ER to improve compliance with care bundles and standardise diagnostic and therapeutic intervention for early identification and fast treatment of septic adult patients. The project was focused on ameliorating sepsis diagnosis in association with an antimicrobial stewardship program based on proper blood cultures (BCs) collection. Specifically, the project was first aimed at identifying the potential septic patients immediately on arrival in the ER with a priority colour code (Fig. 1). This was followed by a standardised diagnostic and therapeutic management of the patient according to the MEWS score (Fig. 2).

To select only those patients with actual systemic infection, thus limiting the heterogeneity of the enrolled population, we included only the patients with positive BCs. For this purpose, we analysed the clinical outcome, defined as in hospital-all-cause mortality of patients accessing our ER with BSI during two different periods of time, from the 1st January 2016 to the 31st December 2017, and from the 1st January 2018 to the 31st December 2019, respectively. The watershed between these two periods was the Sepsis Project implementation.

## Results

In period A 1402 sets of BCs were collected, 29.1% of them were positive while in period B 1060 BC sets were collected, 29.6% were positive (p = ns). Overall, 722 patients admitted in ER had positive BC on admissions, 408 in period A and 314 in period B, and these patients with positive blood culture were included in the present study. Patient’s characteristics in the two periods are summarised in Table 1, both groups were comparable for age, gender, and concomitant diseases, MEWS, and SOFA score.

**Table 1:** Patients’ general characteristics.

	Period A	Period B	*p*
Age	76 (65–84)	76 (67–84)	0.6
Female	188 (46%)	220 (54%)	0.8
Male	142 (45%)	172 (55%)	
SOFA	3 (2–4)	3 (2–5)	0.13
MEWS	4 (3–6)	4 (3–5)	0.11
HIV	5 (1.23%)	2 (0.64%)	0.4
CKD	35 (8.58%)	9 (2.87%)	0.001
DM	72 (17.65%)	61 (19.43%)	0.5
Neoplasms	67 (16.42%)	41 (13.06%)	0.2
CHD	36 (8.82%)	23 (7.32%)	0.5
ICU stay	43 (10.5%)	29 (9.2%)	0.5
Time to BCs collection	3.3 (1–5)	1.6 (0.5–4)	0.04
BCs turnaround time (hours)	12.4 (10.1–17.1)	12.4 (10–17.5)	0.8
Appropriated antimicrobial treatment	266 (65.2%)	249 (80.6%)	< 0.001
Antibiotic treatment started within			
1 h	27 (8.8%)	79 (25.3%)	< 0.001
3 h	77 (28.4%)	101 (32.4%)	
6 h	70 (27.1%)	62 (19.9%)	
24 h	92 (32%)	70 (22.4%)	
Color code associated mortality			
Green	5 (2.7%)	1 (1.1%)	< 0.01
Yellow	55 (29.8%)	30 (15.2%)	
Red	17 (43.8%)	9 (40.9%)	

*SOFA *sequential organ failure assessment, *MEWS *modified early warning score, *HIV* human immunodeficiency virus, *CKD* chronic kidney disease, *DM* diabetes mellitus, *ICU* intensive care unit, *CHD* chronic hepatic disease, *BCs* blood cultures.

BC turnaround time was 12.4 h (10.1–17.1) in period A and 12.4 h (10–17.5) in period 2 (p = 0.8).

Microbiology data are summarized in Table 2.

**Table 2:** Bacteria isolated in blood cultures.

	Period A	Period B
Total bacteria isolated	459	366
GN bacteria	258 (56.2%)	218 (59.5)
* Escherichia coli WT*	130 (28.32%)	101 (27.60%)
* Escherichia coli ESBL*	36 (7.84%)	29(7.92%)
* Klebsiella pneumoniae WT*	23 (5.01%)	16 (4.37%)
* Klebsiella pneumoniae ESBL*	4 (0.87%)	7(1.91%)
* Klebsiella pneumoniae KPC*	4 (0.87%)	1 (0.27%)
* Klebsiella* spp.* WT*	5 (1.09%)	8 (2.19%)
* Klebsiella* spp.* ESBL*	1 (0.22%)	
* Proteus mirabilis*	13(2.83%)	14(3.83%)
* Enterobacter WT*	6(1.31%)	8 (2.19%)
* Enterobacter EBSL*	1 (0.22%)	1 (0.27%)
* Salmonella *spp	5 (1.09%)	2 (0.55%)
* Serratiamarcescens WT*	2 (0.44%)	1 (0.27%)
* Citrobacter* spp.	2 (0.44%)	1 (0.27%)
* Providencia *spp* WT*	2 (0.44%)	
* Providencia *spp* ESBL*	1 (0.22%)	
* Hafnia alvei WT*	1 (0.22%)	
* Shigella* spp	1 (0.22%)	
* Pseudomonas aeruginosa*	13 (2.83%)	17 (4.64%)
* Pseudomonas *spp	2 (0.44%)	1(0.27%)
* Acinetobacter baumanii*		1 (0.27%)
* Acinetobacter* spp	1 (0.22%)	2(0.55%)
* Neisseria meningitidis*	1 (0.22%)	1 (0.27%)
* Haemophilus influenzae*	1(0.22%)	1(0.27%)
* Stenotophomonas maltophilia*	1 (0.22%)	1 (0.27%)
* Leclercia adecarboxylata*	1 (0.22%)	
* Morganella morganii*	1 (0.22%)	
* Achromobacter xylosoxidans*		2 (0.55%)
* Shewanella putrefaciens*		1 (0.27%)
* Aeromonas caviae*		1 (0.27%)
* Bordatella holmesii*		1 (0.27%)
GP bacteria	189 (41.2)	135 (36.9)
* Staphylococcus aureus MS*	45 (9.8%)	29 (7.92%)
* Staphylococcus aureous MR*	17 (3.7%)	8 (2.19%)
* Coagulase-negative Staphylococcus MS*	12 (2.61%)	8 (2.19%)
* Coagulase-negative Staphylococcus MR*	20 (4.36%)	7 (1.91%)
* Streptococcus pneumoniae*	24 (5.23%)	23 (6.28%)
* Streptococcus gallolyticus*	5 (1.09%)	7 (1.91%)
* Streptococcus anginosus*	5 (1.09%)	6 (1.64%)
* Streptococcus agalactiae*	4 (0.87%)	3 (0.82%)
* Streptococcus constellatus*	6 (1.31%)	1 (0.27%)
* Streptococcus pyogenes*	3 (0.65%)	1 (0.27%)
* Streptococcus viridans*	1 (0.22%)	
* Streptococcus* spp	12 (2.61%)	19 (5.19%)
* Enterococcus faecalis*	19 (4.14%)	12 (3.28%)
* Enterococcus faecium*	3 (0.65%)	6 (1.64%)
* Enterococcus spp.*	5 (1.09%)	2 (0.55%)
* Listeria monocytogenes*	5 (1.09%)	1(0.27%)
* Lactobacillus* spp	1 (0.22%)	
* Bacillus* spp	1 (0.22%)	
* Nocardia* spp.	1 (0.22%)	
* Aerococcus urinae*		1 (0.27%)
* Corynebacterium spp.*		1 (0.27%)
* Anaerobes*	8(1.74%)	12(3.28%)
* Candida* spp	4 (0.87%)	1 (0.27%)

*GP* Gram positive, *GN* Gram negative, *WT* wild type, *ESBL* extended spectrum beta-lactamase, *MS* methicillin sensitive, *MR* methicillin resistant.

Gram-negative (GN) bacteria were the principal isolated pathogens (in 56.2% in period A and 59.5% in period B) with Enterobacterales being responsible for 51.6% of BSI episodes in both period A and B. The rate of extended-spectrum beta-lactamase (ESBL) producers was roughly 10% and was stable among the 2 periods.

Gram positive (GP) bacteria were documented in 41.2% and 36.9%% of patients in period A and B respectively (being *Staphylococcus aureus* and *Streptococcus pneumonia*e the most represented). Polymicrobial BSI was recorded in 10.3% and 11.8% in periods A and B, respectively. The bacterial species were equally distributed among the 2 periods (p = 0.5).

Among patients included in the present study, in-hospital mortality was 18.9% in period A and 12.7% in period B (p = 0.03). At multivariable analysis, mortality was reduced in period B compared to period A (OR 0.64, 95% CI 0.41–0.98, p = 0.045). Having an infection due to GP bacteria or polymicrobial was associated with an increased risk of death, as it was having a neoplasm or diabetes (Table 3).

**Table 3:** Factors associated with the risk of death in univariate and multivariate analysis.

	Univariate analysis		Multivariate analysis	
	OR (95% CI)	P value	OR (95% CI)	P value
Age (per 1 year)	1.04 (1.04–1.11)	0.000	1.04 (1.02–1.05)	< 0.001
GP bacteria versus GN bacteria	2.01 (1.31–3.09)	0.001	2.47 (1.57–3.9)	< 0.001
Polimycrobial versus GNB	1.86 (0.99–3.49)	0.052	1.95 (1.01–3.74)	0.45
Neoplasm	2.06 (1.27–3.35)	0.003	2.06 (1.24–3.42)	0.005
DM	0.42 (0.21–0.79)	0.007	0.4 (0.2–0.8)	0.007
Period B versus A CKD	0.63 (0.41–0.95) 0.39 (0.40–10.1)	0.027	0.64 (0.41–0.98)	0.04

*DM* diabetes, *GP* Gram positive, *GN* Gram negative, *CKD* chronic kidney disease, *DM* diabetes mellitus, *CKD* chronic kidney disease.

Interestingly, we noticed a significant reduction in mortality according to the colour code of admission. In particular, mortality in patients admitted with the yellow code was 29.5% in period A versus 15.2% in period B (Table 1).

Adequate antimicrobial treatment was observed in 65.2% of patients in period A and in 80.6% in period B (p < 0.001). The timing of antimicrobial therapy has been reported in Table 1, and there were statistically differences between the two periods (p < 0.001). Neither timing of antimicrobial therapy nor receiving adequate therapy were associated with reduced mortality, possibly because the study was not powered on these secondary endpoints. Twenty-nine patients (9.2%) were admitted in ICU during period B. Age and having a GP BSI were associated with ICU admission. One out of 29 patients admitted to ICU did not receive adequate antimicrobial therapy in ER. Timing of antimicrobial therapy was not associated with ICU admission (Table 4).

**Table 4:** Factors associated with the risk of ICU stay in univariate and multivariate analysis.

	Univariable analysis		Multivariable analysis	
	OR (95% CI)	P	OR (95% CI)	p
Periodo B versus period A	0.86 (0.53–1.42)	0.56	0.86 (0.53–1.48)	0.661
Age	0.97 (0.96–0.98)	< 0.001	0.97 (0.96–0.98)	0.000
GPB versus GNB	2.8 (1.21–6.5)	0.02	1.34 (0.79–2.28)	0.279

*GPB* Gram-positive bacteria, *GNB* Gram-negative bacteria.

To evaluate how the project affected the outcome of all patients with suspect of sepsis, the mortality in patients who collected blood cultures (1402 and 1060 in the two periods) was evaluated and was 13.8% and 11.1%, in period A and B respectively (p = 0.0063). To evaluate the impact of the antimicrobial use on hospital microbial ecology in the two periods, *Clostridium difficile* colitis were compared. No difference was observed in cases of *Clostridium difficile colitis* in the two periods (220 in period A and 232 in period B, p = ns). Moreover the incidence of *Enterobacteriaceae carbaenemase resistant* (infection/colonization) in the two period incidence was stable (379 in period A and 287 in period B, p = ns).

## Discussion

After the implementation of a sepsis project based on the bundles as in the Surviving Sepsis campaign in the ER of our Hospital, a reduction in in-hospital mortality of patients with documented BSI associated with signs or symptoms of sepsis was observed.

Although this result is in line with other studies^13^, some scepticism was also observed ^14,15^. As a result, the real advantages of sepsis bundles have been questioned, and uncertain data are currently available.

The main feature of our study has been the inclusion of those patients with positive BC, rather than all those with a clinical suspicion of sepsis, as the sepsis clinical presentation might frequently resemble that of other morbid conditions^16^. Although we agree with Fuchs et al.’s findings that a standardised method of BCs may positively contribute to optimised management of sepsis, and consequently to an improved survival rate of septic patients^14^, it should be mentioned that BCs collection procedures have been already standardised during period A in our ER. This virtuous practice, which had already been introduced in the period A, and consistently maintained throughout the period B, allowed to attain a positive BCs rate around 30%. Since a BCs low yield may lead to prolonged hospitalisation, and broad antibiotic usage^17^, ours is undoubtedly a remarkable result.

The availability of an antimicrobial stewardship program allowed the setting of appropriate treatment in a significant slice of patients (80%) within 24 h. A major advantage for the timely provision of proper antimicrobial treatment, was the short turn-around time of BCs (12.4 h), which allowed the ID specialists to quickly acquire the necessary data to prescribe a proper therapy. However, concerning the timely administration of antimicrobial therapy within one to six hours from admission, our study failed in finding a significant association between the timely antimicrobials administration and favorable clinical outcome, consistently with others^18,19^, perhaps also because the study was not powered on this secondary objective. Patients with yellow code on admission had the greatest benefit in terms of mortality. This data is intriguing, as it is the most frequently observed presenting pattern in clinical practice, where the application of the bundle finds a more prominent rationale. Possibly, in these patients, a prompt antibiotic therapy might have a more significant impact on septic shock patients’ outcomes^20^. Moreover, our nurses' and physicians’ attention to sepsis significantly increased, consequently reducing their operating variability. This certainly played a key role, underlying the importance of standardising clinical practice.

In our view, since no standardised guidelines are applicable to every healthcare setting, it is of paramount value that each hospital develops and closely adheres to its own internal procedures, which best reflect its own internal organisation. Furthermore, reporting the obtained results help comparing the different strategies. Finally, regarding the microbiological aetiology of the observed BSI, the higher death rate associated with GP rather than GN microorganisms in our analyses, is not universally recognized^21^. However, over recent years, multidrug-resistant patterns in GP bacteria have resulted in difficult-to-treat infections, which in turn caused a current, overall increase of mortality^22^. Moreover, *Staphylococcus aureus* and coagulase-negative staphylococci, which were mostly isolated in our patients, are the leading cause of serious infections, such as endocarditis, with, furthermore, an increasing, high rate of methicillin resistance. Our results confirm the opinion that, although recent global attention has focused on the issue of multidrug resistance (MDR) in GN bacteria, GPs are also a serious concern.

Our study has several limitations. Firstly, it is a single-centre setting, with a relatively small number of patients and secondly, due to its retrospective nature. Moreover, the implementation of the Sepsis Project was associated with a mortality improvement, rather than being representative of causal factors. There is uncertainty about the mortality benefit because it cannot be definitively stated if this is due to increased physicians' and nurses’ awareness of severe sepsis, the Sepsis Project implementation, or other, unrelated determinants.

However, in summary, the difference in terms of saved human lives between period A and period B, pre and post Sepsis Project implementation respectively, has been overwhelming, and we can assume that this result came from an overall improvement in sepsis management and establishing a quality pathway, from the ER front door toward ward-admission, which we believe that it’s crucial to report.

## Methods

### Data source and study design

This is a retrospective observational study, conducted at our Hospital, Fondazione IRCCS Policlinico San Matteo of Pavia, Northern Italy. The study was approved by the local Research Ethics Committee Foundation (P-20200109218, prot 20210015825).

All patients admitted to the ER of our Hospital from the 1st January, 2016 to the 31st July 2019 were identified through electronic records and their medical data were retrospectively collected.

The study comprises of two periods:

Period A: From the 1st Jan 2016 to the 31st Dec 2017 introduction of BC collection in ER 24/24 h.

Period B: From the 1st Jan 2018 to the 31st Jul 2019 introduction of the Sepsis project in ER.

### The sepsis project

The aim of the sepsis project implementation in the ER was to standardise diagnostic and therapeutic intervention for early identification and fast treatment of septic adult patients. Due to the knowledge that only a multifaceted approach might be effective, a multidisciplinary team was assembled. It was composed of a medical direction member and one specialist doctor for each of the following fields: emergency medicine, resuscitation and anesthesiology, infectious diseases (ID), and microbiology. A fundamental culture change from the ER staff was the desired objective and this team was first accountable for raising awareness of the existing problem, and the consequent demand for improvement. The specific roles and responsibilities of these specialists have been thoroughly defined and a diagnostic and therapeutic plan has been set up.

The strength of the sepsis project has been to combine practical and educational interventions.

Practically, a “sepsis pathway” has been designed, to identify the potential septic patients immediately on arrival in the ER, as it happens for stroke and myocardial infarction. This “on door” identification has been conceived to minimise the waste of valuable time. In more detail, a triage nurse collects a brief anamnesis and firstly assigns to the patient a priority colour code, according to the MEWS score (Fig. 1).

A MEWS score ≥ 3 with signs or symptoms of infections prompts a yellow priority code and led directly to the ER evaluation box, where a combined nurse and physician’s intervention has been organized as follows. A nurse, assigned to the examination room, reassesses the patient’s vital signs, finds proper venous access, and performs laboratory tests and 2 sets of BCs. A physician visits the patient and prescribes treatments (fluids, antimicrobial therapy, dopamine if necessary), alerts, if necessary, other consultants (resuscitation and anesthesiology and/or ID specialists,) and transfers the patient to the most suitable department.

The color code included the follows criteria: red color when the MEWS score is ≥ 9 and the patient is in immediate risk for life, yellow code for MEWS ≥ 3 and < 9, green code for MES < 3.Moreover, requiring blood tests have been simplified by generating sepsis-specific panels and a summary algorithm has been designed. Particularly, the “First Hour- Sepsis six” bundle is referred to the 3 diagnostic (take three) and 3 therapeutic (give three) steps to be delivered by staff within 1 h (Fig. 2).

Specific training of the nurses has been focused on the correct BCs pre-analytical phase, and the automated BC system BacT/ALERT (bioMérieux SA, Marcy-l’Etoile, France) had been placed in the ER^23^.

Workshops and conferences have been organised and conducted every week for a year, focusing on the recognition, monitoring, and management of septic patients. To ensure that improvements were sustainable in the long term, recruitment of new members of the team had followed a similar practice. To achieve greater staff compliance, minimising potential oversights, poster formats of the Sepsis bundles algorithm were displayed in all the ER areas.

### Blood cultures

BCs were processed as previously described^15^. Positive BCs were defined according to Weinstein ^8^. Coagulase-negative staphylococci, aerobic and anaerobic diphtheroids, *Micrococcus* spp., *Bacillus* spp., and viridans streptococci were considered contaminants if only one bottle was positive, and susceptibility testing was not performed.

An alert system was implemented in case of positive BCs that consisted of a phone call to the ward in charge of the patient and e-mail was automatically sent to the ID consultant physicians.

The organisms are identified through Gram stain on smears and by Matrix-Assisted Laser Desorption Ionization time-of-flight (MALDI-TOF) (Bruker Daltonics GmbH, Bremen, Germany) or by biochemical tests using Phoenix 100 (BD) automated system N-MIC/ID or P-MIC/ID panel GmbH, Bremen, Germany). The antimicrobial susceptibility is tested using Phoenix 100 (BD).A real-time PCR technology GeneXpert system (Cepheid, Sunnyvale, CA), performed according to the manufacturer's instructions, is used to detect methicillin/oxacillin resistance for BCs growing Gram-positive (GP). A multiplex nested PCR FilmArray enables rapid (1 h) and accurate detection of 24 pathogens (bacteria and yeasts) and 3 antibiotic resistance genes and rapid immune-chromatographic tests are used for the determination of resistance markers.

### Antimicrobial stewardship

The ER staff has been taught to consult an on-call 24 h a day ID specialist to set the most appropriate antimicrobial regimen, once microbiological results are available. Besides, online internal guidelines have been provided for empirical treatment of lung, urinary tract, abdominal and skin and soft tissue infections.

For carbapenems, linezolid, tigecycline, and daptomycin and 4th generation cephalosporin an ID consultation was considered mandatory.

According to the in vitro sensitivity of the isolated microorganisms, an empirical treatment has been considered appropriate if started within the first 24 h from admission, if effective according to the in vitro results, adequate to the site of infection, and administered at the appropriate dose and schedule.

To evaluate the impact of antimicrobial use in hospital microbial ecology Clostridium difficile infection registered in the two periods were compared. No difference was observed in cases of Clostridium difficile infection in the two periods (220 in period A and 232 in period B). Moreover the incidence of Enterobacteralescarbapenemase resistant (infection/colonization) in the two period incidence was stable (379 in period A and 287 in period B).

### Inclusion criteria and patients’ characteristics

All the patients who fulfilled the following inclusion criteria were then recruited into the study:

- Patients with suspect of sepsis with a MEWS score ≥ of 3 upon admission in the ER and
- positive BC(s).

### Variables

The available data for both the considered periods included demographics (sex and age), co-morbidities (cancer, diabetes, hypertension, chronic kidney disease and chronic liver disease), and microbiological data (type of microorganism causing the infection with specific susceptibility profiles) and the severity scores (Sequential Organ Failure Assessment [SOFA] and modified early warning scoring [MEWS])^24^. Differently, appropriateness and timing of antimicrobial therapy were retrospectively collected only for period B.

### Outcomes of the study

The primary outcome of the study was in-hospital all-cause mortality. The study aimed to evaluate the effects of the sepsis project implementation on patients’ in-hospital all-cause mortality, by comparing mortality rates between the 2 considered periods (both A and B period).

Secondary outcomes included intensive care unit (ICU) admission rate in the two periods. Moreover, the impact of appropriate and timely antibiotic administration (within 1 h, within 3 h, within 6 h and within 24 h since ER admission) on in-hospital mortality and ICU admission was evaluated in period B only.

### Accordance statement

The study protocol was written in accordance with the ethical guidelines of the 1975 Declaration of Helsinki.

### Approval statement

The SePs Study was approved by the Ethical Committee of Fondazione IRCCS Policlinico San Matteo, PAvia (Comitato Etico Pavia, no. prot. P-20200109218, prot 20210015825). All participants have signed an informed consent for data collection.

### Statistical methods

Continuous variables were expressed as a median and interquartile range. Qualitative variables were expressed as they absolute value accompanied by a percentage. The median values for the continuous variables from both groups were compared using the Mann–Whitney U test. Proportions were compared using the chi-squared test. To analyze the difference in mortality between the two periods, an univariate and multivariate logistic regression was used. The risk of in-hospital mortality was expressed as an odds ratio (OR) and a 95% confidence interval (95% CI). The multivariate analysis included the variables that demonstrated differences with a p < 0.1 in the univariate analysis.

## Data Availability

The datasets generated and analysed during the current study are not publicly available, because they were from the electronic records of patients hospitalized in IRCCS Policlinico San Matteo. Data supporting the findings of this study are available from the corresponding author (M.C.) on request.
